# X-ray diffraction imaging of fully packaged n–p–n transistors under accelerated ageing conditions

**DOI:** 10.1107/S1600576722007142

**Published:** 2022-08-30

**Authors:** Brian K. Tanner, Andreas Danilewsky, Patrick J. McNally

**Affiliations:** a Department of Physics, Durham University, South Road, Durham DH1 3LE, United Kingdom; b School of Electronic Engineering, Dublin City University, Dublin, Ireland; c Kristallographie, Albert-Ludwigs-Universität, Hermann-Herder-Straße 5, Freiburg D-79104, Germany; SLAC National Accelerator Laboratory, Menlo Park, USA

**Keywords:** X-ray diffraction imaging, current crowding, thermal dilation, silicon devices

## Abstract

X-ray diffraction imaging was used to monitor the local strains that developed around individual n–p–n bipolar transistors within fully encapsulated packages under conditions of extremely high forward bias to simulate accelerated ageing. Die warpage associated with the packaging was observed to relax systematically as the polymer became viscous due to the temperature rise associated with the dissipation of heat in the transistor. The direct image size and intensity from the individual transistors were interpreted in terms of a model in which local thermal expansion is treated as a cylindrical inclusion of distorted material, contrast arising principally from lattice tilt. The extension of the thermal strain image along the emitter with increasing power dissipation was ascribed to the effect of current crowding in the emitter region. Weaker large-area contrast associated with the base–collector region was interpreted as arising from the smaller change in effective misorientation at the high X-ray energy of thermal lattice dilation in the base region.

## Abstract 2

X-ray diffraction imaging contrast from individual silicon n–p–n bipolar transistors within fully encapsulated packages under conditions of extremely high forward bias is interpreted as arising from the effects of current crowding in the emitter region.

## 1. Introduction

Integrated circuit chip packaging plays an important role in allowing manufacturers to stay aligned to Moore’s law, which predicts that the number of transistors in an integrated circuit doubles roughly every two years (Moore, 1965 ▸). While semiconductor manufacturers continue to miniaturize device structure dimensions (‘more Moore’), many of the disruptive advances use technologies broadly described as heterogenous integration. Indeed, the IEEE publishes its own quasi-annual heterogenous integration roadmap (Chen & Bottoms, 2019 ▸) with inputs from major academic and industrial stakeholders in this area. One can broadly define heterogenous integration as the placement of manufactured chips into a higher-level system, an example of which includes system in package structures in which a collection of integrated circuits is enclosed in a single carrier package. Recent advances in chiplet technology require individual die to be manufactured with very precise structures so they can be linked together, like ‘LEGO bricks’, on what is known as an interposer. This is essentially an interface ‘bus’ electrically linking one chiplet to another inside a full system package (Li *et al.*, 2020 ▸). These different structures are becoming very complex, involving the interplay of many dozens of materials. The physical and thermomechanical properties of each of these have to be understood in terms of the role each material plays in the completed package. However, the most important components therein remain the individual transistor devices, and means of *in situ* and *operando* monitoring of their performance are important for future design and reliability studies.

Following the pioneering work of Toda & Ikarashi (2010 ▸), we have shown that high-energy X-ray diffraction imaging (XRDI) can be used successfully in the transmission section topography mode to determine the long-range warpage in silicon dies, fully packaged in small outline integrated circuits (SOICs) (Bose *et al.*, 2016 ▸; Tanner *et al.*, 2017 ▸). Though laboratory sources can be used in certain circumstances (Meng *et al.*, 2017 ▸; Tanner *et al.*, 2021 ▸), synchrotron radiation provides much better data sets. Using synchrotron radiation, we demonstrated that XRDI can be used in this section topography mode under white beam conditions to measure the individual long-range warpage of stacked and interconnected chiplets (Tanner *et al.*, 2017 ▸, 2021 ▸).

However, the section topography configuration, with its narrow field of view, is inappropriate for monitoring the strains around individual devices. The present study focuses on full-area white beam imaging of a simpler, but nevertheless fully packaged, SOIC structure containing bipolar n–p–n transistors. (Fully packaged means that the Si chips inside the packages are totally encapsulated and are not opened or perturbed in anyway by these experiments.) Our objective was, in part, to determine the level of warpage across LM3046 devices and also to understand the contrast associated with the local thermal strains resulting from stressing the individual bipolar transistors under accelerated ageing conditions. In bipolar junction transistors, the phenomenon of current crowding occurs as the current density increases and is observed as a non-homogenous distribution of current close to the periphery of the regions where the base and emitter semiconductor regions come into contact (Sze & Ng, 2007 ▸). Imaging of this effect in silicon does not seem to have yet been achieved. Although monochromatic imaging appears to be necessary for the identification of strains in multiple-stacked chiplets, understanding the behaviour of packages containing a single die is an important step in progressing to the study of more complex structures.

The lifetime of bipolar devices drops with increasing temperature that arises from power dissipation in the transistor (Toshiba, 2021 ▸). Silicon drive transistors can dissipate substantial energy and 0.5 W is not atypical. Lifetimes fall by a factor of about 2 for every 10 K increase in operating temperature, and short-term failure is likely to result from operation at over 448 K. Though monitoring of the outside of the package can give clues as to the internal temperature, there are some parameters that are difficult to determine from such macroscopic measurements. These include the magnitude of the localized thermal strains associated with electrical overload of individual silicon bipolar junction transistors.

Using high-energy XRDI in white beam mode, we demonstrated that the development of the localized thermal strain around the individual devices inside the fully encapsulated LM3046 chip packages can be monitored (Tanner *et al.*, 2019 ▸). The device temperature associated with these strains can be estimated by simultaneously measuring the external package temperature. Subsequent experiments on the same devices indicate that the dynamic range of the initial experiments was limited, thereby failing to reveal a key contrast feature. In this paper we report a detailed examination of the diffraction contrast in new experiments and make interpretions in terms of emitter current crowding and resistive heating in the junction regions. This appears to be the first time that current crowding has been directly imaged in fully packaged chips under *operando* conditions.

## 2. Experimental

**Figure 1:**
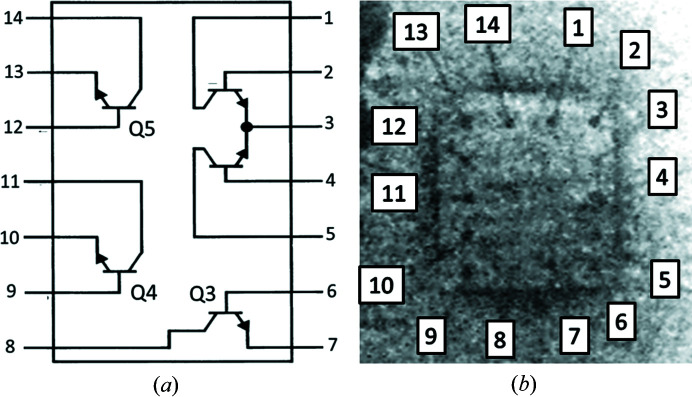
(*a*) Schematic of the positions of the transistors in the LM3046 integrated circuit chip as they appear in the diffraction images. (*b*) Radiograph showing the bond pad locations on the chip as viewed in the diffraction images. Note this is mirrored with respect to the schematics on the manufacturers’ data sheets. Chip dimensions 1 × 1 mm.

**Figure 2:**
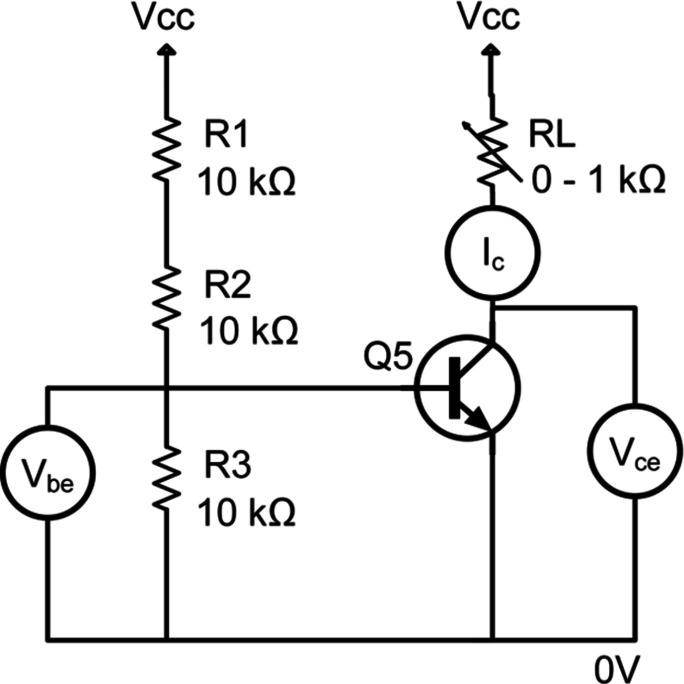
Drive circuit for individual transistors on the LM3046 device.

Wide-beam XRDI experiments were carried out under these conditions on 1.5 mm-thick 14-pin LM3046 SOIC packages (Fig. 1 ▸) containing three individual bipolar n–p–n junction transistors and a differential current mirror pair. The LM3046 transistor array package is commonly used in low-power electronic systems operating in the DC to VHF frequency range. They are used as discrete transistors, but also for instrumentation applications since the transistors on the chip are closely electrically and thermally matched (Texas Instruments, 2017 ▸). Accelerated ageing experiments under conditions of extreme forward bias were undertaken on transistors Q3, Q4 and Q5 of Fig. 1 ▸, using the same circuit configuration as our earlier experiments (Fig. 2;  ▸Tanner *et al.*, 2019 ▸). The power dissipated in the transistor was increased stepwise by varying resistor *R*_L_, allowing thermal stability to be achieved for 10 s between each step. The power transition between steps was approximately linear over a period of 10 s.

X-ray energies in the region of 33 keV were judged to provide a suitable compromise between lack of absorption and intensity of the local image contrast. All data were taken at beamline B16 of the Diamond Light Source, Didcot, UK, under white beam conditions. Diffraction angles were chosen to select X-rays of about 33 keV, which were detected directly by the Photonic Science sCMOS camera which has a 7 mm-diameter field of view, 1920 × 1080 pixels, 14 bit dynamic range and 3.2 µm dimension optical pixels. All experiments were undertaken in standard multi-bunch mode with a machine current of 300 mA. Samples were mounted on a purpose-built breadboard mounted on the optics stage of the general-purpose optical table which supports the imaging cameras. Care was taken to ensure that the image contrast remained within the dynamic range of the detector which was situated 210 ± 5 mm from the sample, its face perpendicular to the incident X-ray beam. The surface temperature of the outside of the package was measured as a function of power through the devices with an FLIR infra-red camera.

The 1 mm square single-crystal wafer in the LM3046 package is cut such that the surface is oriented parallel to (111) with the [110] direction parallel to the edge with five pin-out connections.

## 3. Results

**Figure 3:**
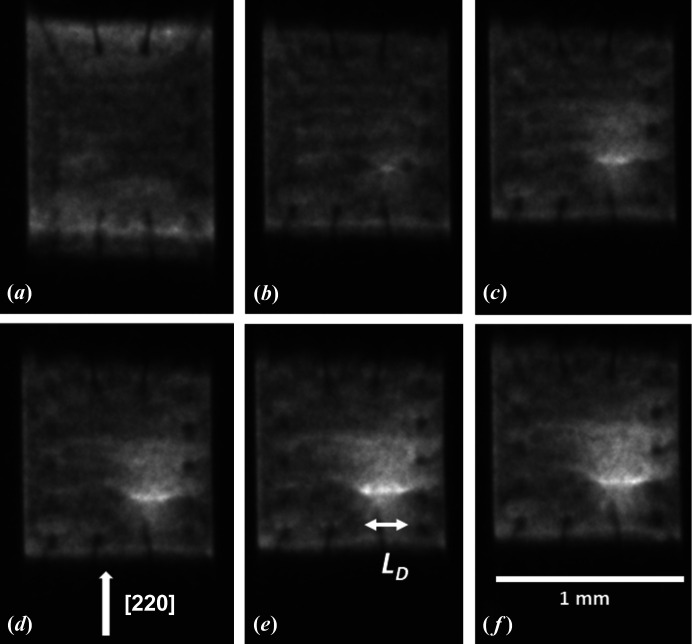
White beam XRDI images taken as the power dissipated in the transistor is incrementally increased by decreasing the value of the resistor *R*_L_. (*a*) *R*_L_ = 800 Ω 0.57 W (*b*) *R*_L_ = 500 Ω 1.32 W, (*c*) *R*_L_ = 300 Ω 1.82 W, (*d*) *R*_L_ = 200 Ω 2.08 W, (*e*) *R*_L_ = 100 Ω 2.32 W, (*f*) *R*_L_ = 50 Ω 2.44 W; 220 reflection, X-ray wavelength 0.038 nm.

X-ray diffraction images, taken in the symmetric 220 reflection, as a function of increase in power dissipated in transistor Q3 are shown in Fig. 3 ▸. At low power dissipation, the diffraction image contrast did not change from that of the unpowered device [Fig. 3 ▸(*a*)]. At 1.32 W [Fig. 3 ▸(*b*)] a direct image of enhanced intensity was observed in the vicinity of the centre of the device. The apparent length of the whole 1 mm die also reduced markedly in the direction of the diffraction vector, and this decrease continued as the power dissipation increased. With increasing power dissipation, the localized direct image increased in length *L*_D_ perpendicular to the diffraction vector direction [Fig. 3 ▸(*c*)]. There was further increase [Fig. 3 ▸(*d*)–3(*f*)] until failure after being held at 2.44 W for times varying from tens of seconds to a few minutes.

**Figure 4:**
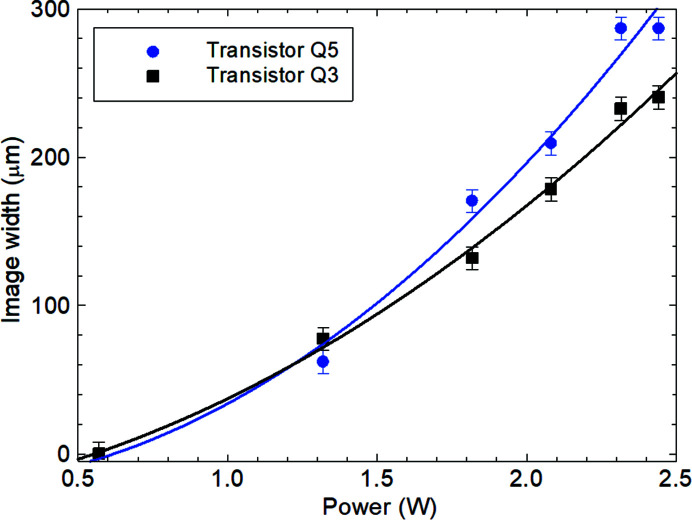
Localized direct image length *L*_D_ as a function of power dissipated. Quadratic fit to the data. 220 reflection.

**Figure 5:**
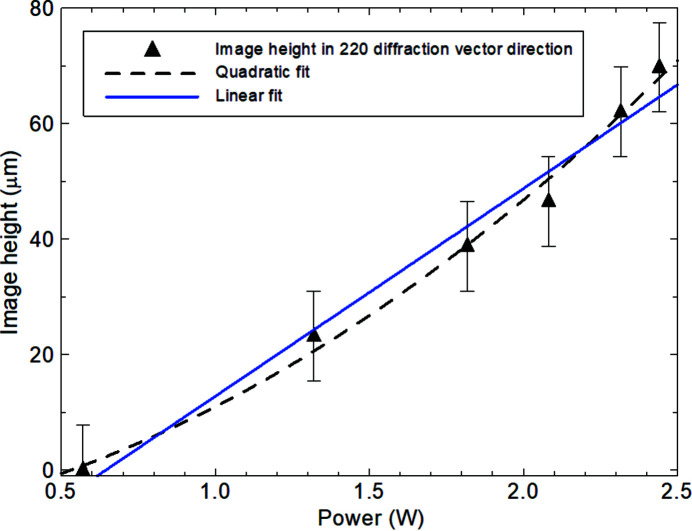
Localized image height in the diffraction vector direction as a function of power. 220 reflection.

**Figure 6:**
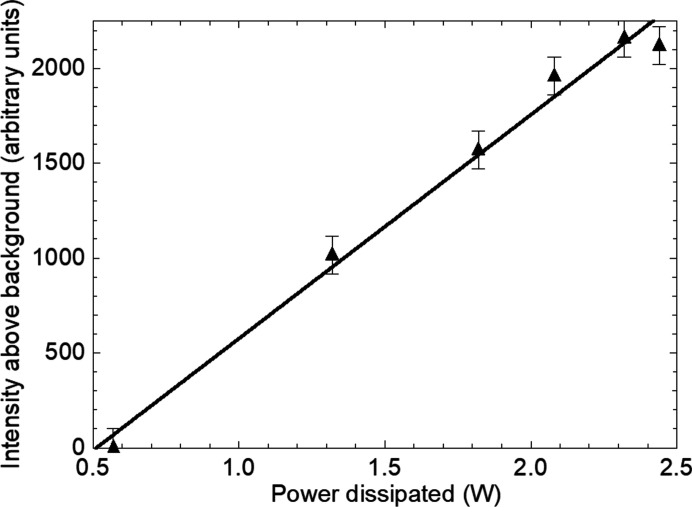
Extra diffracted intensity above background for the direct image hot spot in Fig. 3 ▸.

As reported in our previous paper (Tanner *et al.*, 2019 ▸), the length of the direct image *L*_D_ varied quadratically with power dissipated for all the devices measured. Though there was a substantial linear component to the curves, in all cases inclusion of the quadratic term was required to accommodate the measurement error bars. As illustrated in the comparison between transistors Q3 and Q5 from different chips in Fig. 4 ▸, there is variation between devices outside of the measurement error. The image height parallel to the diffraction vector also increased, but by less than the increase of the width perpendicular to the diffraction vector (Fig. 5 ▸). While a quadratic fit to the data yielded a correlation coefficient of 0.992 compared with that for a linear fit of 0.977, the error bar size is such that caution should be exercised in claiming the presence of the quadratic term. Within the precision of measurement, the ratio of the image length to image height remained constant at 3.5 ± 0.3. The maximum intensity in the image hot spot varied approximately linearly with power dissipated in the transistor (Fig. 6 ▸).

**Figure 7:**
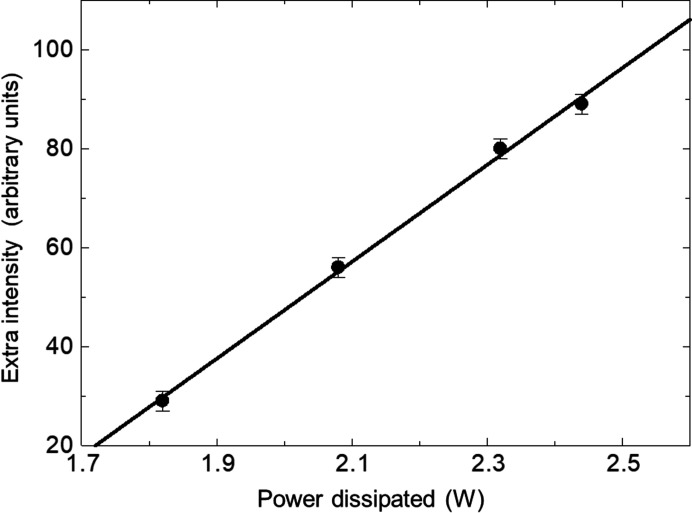
Extra intensity at the centre of the large-area contrast in Figs. 3 ▸(*c*)–3(*f*) as a function of power dissipated.

At 1.82 W, weak contrast associated with the whole device area became visible [Fig. 3 ▸(*c*)–3(*f*)]. This feature was not identified in our previous experiments owing to the lower contrast in the images but was found for all the transistors examined here. Further increase in power dissipation was accompanied by enhancement of the contrast from the large area, the excess intensity above the diffracted intensity from the main body of the crystal varying linearly with power dissipated (Fig. 7 ▸). The area over which the contrast was observed did not change in dimension with increasing power dissipation.

**Figure 8:**
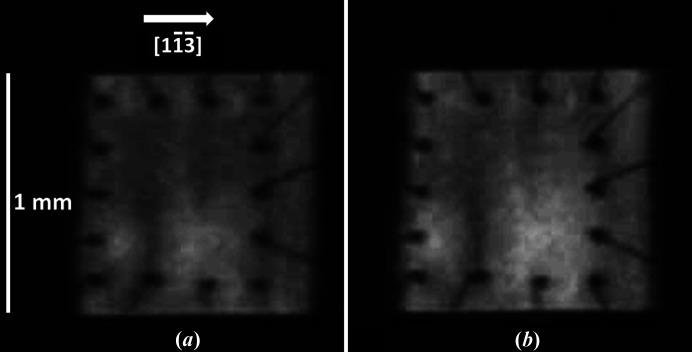
Images, taken with the 11 3 reflection of transistor Q3 at (*a*) 1.4 W and (*b*) 2.08 W. The region of contrast close to the chip edge, at the bottom left adjacent to pin 9, is an artefact on the camera. It does not show strongly in Fig. 3 ▸ owing to the higher contrast of the surrounding features in the 220 reflection.

With the sample rotated by 90° about the surface normal and selecting the 11 3 reflection, the contrast was weaker (Fig. 8 ▸) and there is no strong direct image. Area contrast appears, extending over the whole region of the transistor, and the intensity of this image increases approximately linearly with power dissipated in the device.

## 4. Discussion

### 4.1. Die warpage

**Figure 9:**
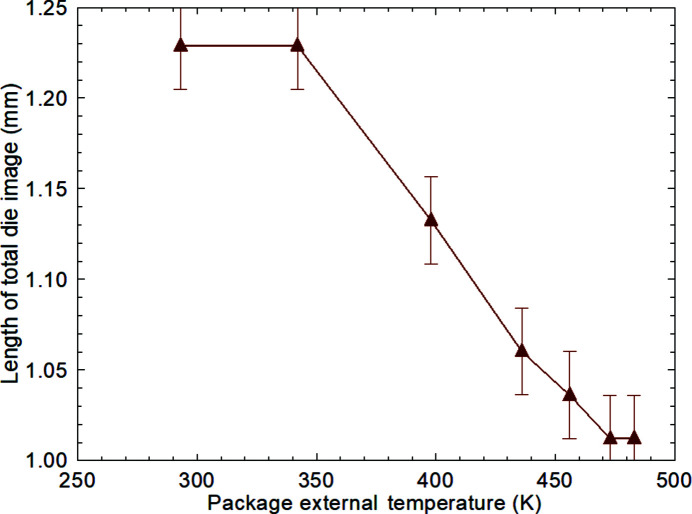
Length of total die image, measured along its left-hand edge, in the projected direction of the diffraction vector as a function of the external temperature of the LM3046 package when power is dissipated in a single transistor.

In Fig. 3 ▸, the X-ray energy was selected as 32.6 keV, giving a diffraction angle of 5.7° for the 220 reflection, and a distortion of [cos(11.4°)]^−1^ = 1.02 exists on the face of the detector set perpendicular to the incident beam. For a 1 mm silicon crystal with zero warpage across it, the image length in the projected direction of the diffraction vector would therefore be expected to be 1.02 mm. At zero and low power, the observed image length of 1.23 ± 0.025 mm accordingly corresponds to a tilt angle of 5.4 × 10^−4^ rad, *i.e.* 111 ± 3′′, across the die. Noting that the sample-to-detector distance of 210 (±5) mm remained unchanged through the sequence of images, Fig. 9 ▸ shows that there is a steady reduction of total image length of the die as the power through the transistor is increased and the package becomes heated. (The outside temperature of the package was found to vary linearly with power dissipated in a single transistor and there was no significant difference in data from the Q3, Q4 and Q5 transistors or from different complete devices.) By a power dissipation of 2.44 W, where the outside package temperature was 483 K, the length of the image corresponds, within measurement precision, to that of an undistorted crystal of 1 mm dimensions.

The data indicate that there is significant strain/warpage locked in by the packaging process when the polymer cools. Such a conclusion is entirely consistent with the findings of our section topography measurements on a number of SOIC packaged silicon dies (Tanner *et al.*, 2017 ▸, 2021 ▸). As the polymer softens above about 373 K (Tanner *et al.*, 2021 ▸) on the rise of the package temperature, the warpage is gradually relaxed. Following failure, the polymer again hardens on cooling to room temperature, reintroducing warpage. On return to 293 K, following failure, the image length was measured to be 1.38 mm, significantly greater than in the as-received chip.

Despite the strain relaxation with polymer softening, even at a device temperature of 423 K, the warpage across the die is still 1.75 × 10^−4^ rad, or 36′′. While this is small compared with the warpage of a fraction of a degree (*e.g.* 5 × 10^−3^ rad) found across many as-packaged devices (Tanner *et al.*, 2017 ▸), it will prove challenging to obtain local images with a monochromated beam at these high energies. The Darwin widths for the silicon 111 and 220 reflections are 1.6 and 1.2′′, respectively, at 32.6 keV, while the sample subtends 4.5′′ at the source.

### 4.2. Image hot spot width in the 220 diffraction direction

In a previous paper (Tanner *et al.*, 2019 ▸), it was noted that the length *L*_D_ of the bright spot in the 220 image (Fig. 3 ▸) increased with power dissipated, and the present results confirm the quadratic dependence of the length with power. Here we argue that this arises from current crowding in the base region below the emitter. At large values of the variable resistor *R*_L_ and low power dissipation in the transistor, no diffraction contrast is visible that can be associated with the electrical operation. The energy deposited in the device is dissipated without sufficiently large lattice expansion in the device region to result in diffraction contrast. At 31 keV X-ray energy, the effective misorientation of the Bragg planes Δ(δθ) (Authier, 2001 ▸), given by

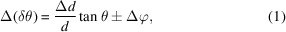

is dominated by the tilt Δφ of the Bragg planes, rather than the dilation Δ*d*, due to the small Bragg angle. When the tilt Δφ > αδω, where δω is the reflection curve width and α is a numerical parameter approximately equal to unity, dependent on the reflection (Miltat & Bowen, 1975 ▸), the region diffracts as a mosaic crystal. (For the 220 reflection in silicon at 31 keV, δω = 1.2′′.) The net intensity contribution of this mosaic region is greater than that of a perfect crystal of the same volume, resulting in the enhanced intensity ‘direct image’ (Authier, 2001 ▸).

The temperature *T* of the device above the external package temperature *T*_0_ can be treated (Tanner *et al.*, 2019 ▸) using the self-heating model of Zweidinger *et al.* (1993 ▸). This results in a thermal resistance *R*_th_ which is inversely proportional to the thermal conductivity and to a geometrical parameter dependent on the width and length of the emitter, the depth of the collector–base junction, and the width of the collector. Then

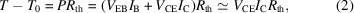

as the base–emitter current *I*_B_ is small compared with the emitter–collector current *I*_C_. *V*_EB_ and *V*_CE_ are the voltage drops across the emitter–base and collector–emitter, respectively, and *P* is the power dissipated.

As the emitter is long compared with its width, and assuming that the thermal expansion of the heated region can be treated as a cylindrical inclusion, the expectation would be that the direct image should appear along the whole length of the emitter, gradually increasing in intensity as the power is increased. The data show that there is an increase in intensity in the image with power dissipated (Fig. 6 ▸), which is consistent with the increase in strain below the emitter region leading to a greater kinematically diffracting volume. Such an increase in local thermal strain also explains the increase in width of the direct image in the direction of the diffraction vector (Fig. 5 ▸) with increasing power. If one models the local thermal strain in the elongated emitter region as a cylindrical inclusion parallel to the **z** direction in the plane of the wafer, the strain tensor components are

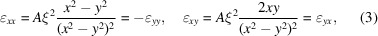

where **x** is in the plane of the wafer and **y** is normal to its surface measured from the centre of the inclusion (Kramer & Bauer, 1967 ▸; Ivanov *et al.*, 1982 ▸; Tanner, 1984 ▸). The parameters *A*ξ^2^ determine the absolute magnitude of the strain field and direct image formation begins when ɛ*_xy_* exceeds approximately 6 × 10^−6^ for the 220 reflection at the X-ray energy used.

**Figure 10:**
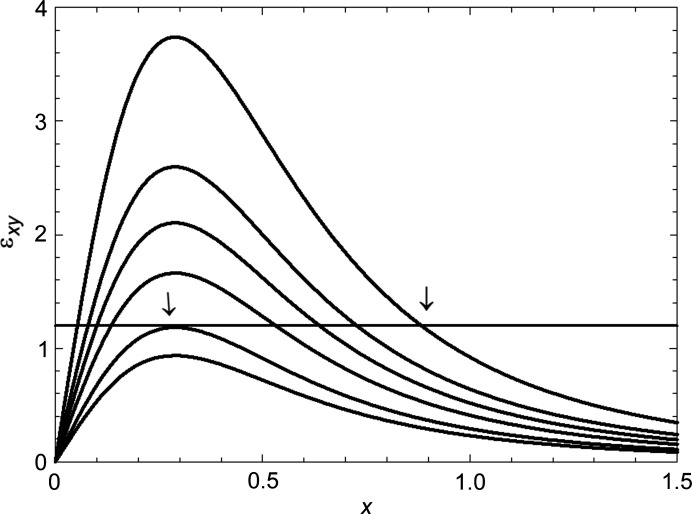
Tilt ɛ_*xy*_ as a function of normalized in-plane coordinate *x*, at fixed depth *y*, for various values of the deformation parameter ξ. From the bottom, curves correspond to ξ = 0.6, 0.675, 0.8, 0.9, 1, 1.2. The value of αδω is set arbitrarily at 1.2 in this example.

**Figure 11:**
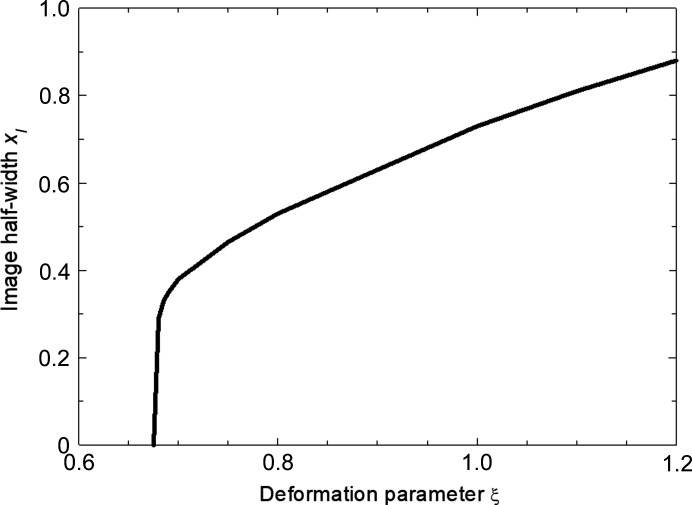
Image formation distance *x*_I_ for a particular depth as a function of deformation parameter *ξ*. Units are arbitrary and normalized.

At a certain depth in the wafer, the lattice plane tilt, given by ɛ_*xy*_, rises rapidly to a maximum before falling away slowly with increasing *x* (Fig. 10 ▸). Where the tilt exceeds αδω, the diffraction image will be formed. As seen in Fig. 10 ▸, for decreasing values of the deformation parameter ξ, the region where the tilt ɛ_*xy*_ exceeds αδω decreases. For values of ξ where the maximum tilt does not exceed this value, no diffraction image is formed [Fig. 3 ▸(*a*)]. The image half-width corresponds to twice the distance at which the image starts to form. Fig. 11 ▸ shows that below a ξ parameter value of 0.675 no image is formed. Over a ξ range from about 0.8 to 1.1, the image half-width *x*_I_ varies approximately linearly with *ξ*. Although the deformation parameter ξ may be taken as proportional to the temperature *T* because of thermal expansion and this steady-state temperature *T* may be expected to vary linearly with power dissipated, there is no direct evidence that the temperature at the emitter is linear with power. However, the almost linear variation of the image width with ξ derived from the model is entirely consistent with the approximately linear variation of the image width in the diffraction vector direction with power which is found experimentally (Fig. 5 ▸).

The increase in intensity of the hot-spot image (Fig. 6 ▸) arises from the increase in volume of the material where the tilt Δφ > αδω. For clarity, the above discussion was restricted to a particular depth, but as the deformation rises with increasing temperature, material at greater depth below the device will reach the tilt threshold. The intensity scales with volume of kinematically diffracting material.

Although the tensor components in equation (3) are only strictly valid for an infinite length defect, to first order, it is evident that the components ɛ_*xz*_ = ɛ_*zx*_ and ɛ_*yz*_ = ɛ_*zy*_ are all zero and hence there will be no tilt of Bragg planes that have a normal lying in the *yz* plane. Therefore, the absence of a hot-spot image in the 11 3 reflection, shown in Fig. 8 ▸, is straightforwardly explicable.

### 4.3. Image hot-spot length perpendicular to the 220 diffraction direction and emitter crowding

The variation in length *L*_D_ of the image in the direction normal to the diffraction vector (Fig. 4 ▸) requires a different explanation. For uniform power dissipation along the emitter region, the entire length of the image should appear at once when the threshold power for image formation is exceeded. There might be a modest increase in length, perhaps comparable to the increase in width discussed above, but the much larger increase in length (Fig. 4 ▸) cannot be explained in this way. The change in length perpendicular to the 220 diffraction vector direction must arise because of a change in the distribution of current, and hence power dissipation, through the device.

**Figure 12:**
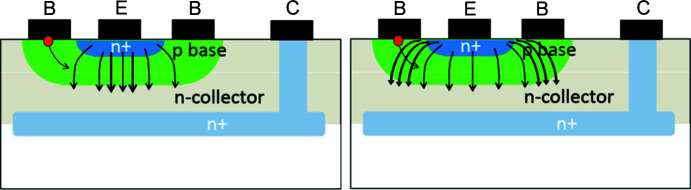
Crowding of the emitter–collector current, the extension of which results in an increase in the length of the direct image associated with the hot spot. (Adapted from https://nanohub.org/resources/5835/download/2009.03.25-ECE606-L28.pdf. Courtesy: Muhammad A. Alam, Purdue University.)

As configured in forward bias, the emitter–collector current is primarily concentrated below the emitter contact at its centre. The increase in current as the voltage across the device is increased results in a localized central hot spot [Fig. 3 ▸(*b*)]. However, there is at the same time an increased emitter–base current. The base region is thin and elongated and has significant resistance (Sze & Ng, 2007 ▸). The emitter–base current flow results in a larger base–emitter potential difference at the edge of the emitter compared with the centre. At high base current, *I*_B_ is effectively confined to two regions of total length *S*_eff_ at the edges of the emitter length *S*, a feature known as ‘current crowding’. This effect has been imaged in light-emitting III-V devices, where it has a major impact on light-emitting diode performance (Malyutenko *et al.*, 2001 ▸, 2015 ▸). For absolute temperature *T*, Hauser (1964 ▸) provides equations governing the variation of *S*_eff_ as a function of *I*_B_/*kT*, where *k* is the Boltzmann constant. The solution shows that *S*_eff_ falls initially slowly, but subsequently rapidly, with increasing *I*_B_. The non-uniform potential difference between emitter and base results in the emitter–collector current being similarly crowded towards the emitter edges, as illustrated in the sketch of Fig. 12 ▸. Hauser (1964 ▸) provides an analytic solution for the emitter–collector current distribution in a double base contact planar bipolar transistor, assuming that the emitter–base junction is an equipotential, but that quantitative analysis is only valid for low-injection current density.

As the emitter–collector current *I*_C_ is substantially greater than the base current, the emitter–collector current dissipates the majority of the heat within the device. Crowding of the emitter–collector current results in heating towards the edge of the collector area, extending the hot spot as the power dissipated increases. [Although the increase in temperature counters the current crowding to some extent, as the temperature *T* is in kelvin, the change is estimated to be only a factor of two (Tanner *et al.*, 2019 ▸) and its impact is less than the increase in *I*_B_ and *I*_C_.] The increase in the length of the hot spot results directly in an extension of the direct image observed [Figs. 3 ▸(*b*)–3(*f*)] in the diffraction image.

### 4.4. Area contrast at the device

The area contrast noted in Figs. 3 ▸ and 8 ▸ appears to be associated with the whole of the base–collector area. While the contrast appears at approximately the same value of *I*_C_ as the hot spot at the emitter contact, it is much weaker. Heating will occur as a result of electrons diffusing through the base being accelerated in the electric field associated with the base–collector voltage *V*_BC_ (∼*V*_CE_). Assuming that the heating is primarily in the thin-base region, direct image contrast will arise primarily from the dilation, which has already been noted to have a relatively small impact on the effective misorientation Δφ, giving rise to weaker contrast. This, however, is visible in both 220 and 11 3 reflections. There is very little tilt associated with such distortion.

The intensity varies linearly with power dissipated (Fig. 7 ▸). Assuming a constant thermal resistance *R*_th_, the temperature rises linearly with power dissipated. As the junction region is very thin compared with the silicon wafer, the top surface may be regarded as the heat source, resulting in a linear temperature gradient through the sample. As the surface temperature rises, the volume of material dilated above the value to cause formation of the direct image increases linearly. Hence there is a linear variation of the direct, kinematically scattering image, as a function of power. Formation of the image due to thermal dilation of the lattice explains its presence in both 220 and 11 3 reflections.

Kirk (1962 ▸) showed that, at high current densities in bipolar transistors, the neutral base region spreads into the collector region owing to build-up of mobile carrier space-charge density in the collector transition layer. Poon *et al.* (1969 ▸) subsequently solved Kirk’s equations numerically to show that, as the injection current increased, the region of maximum electric field moved from the transition region between base and collector to the bottom of the epitaxial collector region. There is no evidence, however, in the diffraction images derived from thermal dissipation for the Kirk effect, probably due to the small thickness of the epitaxial junction region.

### 4.5. Wider significance

The warpages measured appear to be significant, especially in an era where the silicon dies are becoming ever thinner, typically 25–100 µm, and encapsulated in packages with ever slimmer profiles. For example, strains induced by such warpage can have a significant impact on the delamination of bonding layers within the chip package (Tsai *et al.*, 2021 ▸). In addition, local strains on the order of 10^−4^, which would be approximately the same order as the warpage measured here, can lead to severe problems with shifts in bipolar junction current gains (beta). These shifts can be on the order of 5% for strains of approximately 6 × 10^−4^ (Jaeger *et al.*, 2013 ▸), which would make them unusable for operational amplifiers in instrumentation-related applications. It is impossible to measure the maximum local strain from the X-ray images presented in this paper, but local strains of the above magnitude are readily found around structural defects in other X-ray diffraction imaging experiments.

## 5. Conclusions

White beam, high-energy X-ray diffraction images show distinctive direct image contrast associated with the thermal effects of current crowding at the emitter–base junction in fully packaged LM3046 n–p–n bipolar junction transistors driven under extreme forward bias *operando* conditions. The main contrast features can be explained using a model of the thermal expansion treated as a cylindrical inclusion of distorted material. Weaker large-area contrast associated with the base–collector region can be interpreted as arising from the smaller effects of lattice dilation at high X-ray energy. Though locked-in warpage from packaging relaxes significantly as the polymer softens on heating, monochromatic mode imaging in order to separate images from multiply stacked devices will prove challenging.
